# Efficient disruption of bcr-abl gene by CRISPR RNA-guided FokI nucleases depresses the oncogenesis of chronic myeloid leukemia cells

**DOI:** 10.1186/s13046-019-1229-5

**Published:** 2019-05-28

**Authors:** Zhenhong Luo, Miao Gao, Ningshu Huang, Xin Wang, Zesong Yang, Hao Yang, Zhenglan Huang, Wenli Feng

**Affiliations:** 1 Department of Clinical Hematology, Key Laboratory of Laboratory Medical Diagnostics Designated by the Ministry of Education, Chongqing Medical University, No.1, Yixueyuan Road, Chongqing, 400016 China; 2 Department of Laboratory Medicine, The First Affiliated Hospital of Chongqing Medical University, Chongqing, 400016 China; 3 Department of Clinical Laboratory, The Children’s Hospital of Chongqing Medical University, Chongqing, 400016 China; 4 Department of Hematology, The First Affiliated Hospital of Chongqing Medical University, Chongqing, 400016 China

**Keywords:** Chronic myeloid leukemia, RNA guided-FokI nucleases, Bcr-abl, Homology-directed repair, Leukemogenesis

## Abstract

**Background:**

The bcr-abl fusion gene encodes BCR-ABL oncoprotein and plays a crucial role in the leukemogenesis of chronic myeloid leukemia (CML). Current therapeutic methods have limited treatment effect on CML patients with drug resistance or disease relapse. Therefore, novel therapeutic strategy for CML is essential to be explored and the CRISPR RNA-guided FokI nucleases (RFNs) meet the merits of variable target sites and specificity of cleavage enabled its suitability for gene editing of CML. The RFNs provide us a new therapeutic direction to obliterate this disease.

**Methods:**

Guide RNA **(**gRNA) expression plasmids were constructed by molecular cloning technique. The modification rate of RFNs on bcr-abl was detected via *Not*I restriction enzyme digestion and T7 endonuclease 1 (T7E1) assay. The expression of BCR-ABL and its downstream signaling molecules were determined by western blotting. The effects of RFNs on cell proliferation and apoptosis of CML cell lines and CML stem/progenitor cells were evaluated by CCK-8 assay and flow cytometry. In addition, murine xenograft model was adopted to evaluate the capacity of RFNs in attenuating the tumorigenic ability of bcr-abl.

**Results:**

The RFNs efficiently disrupted bcr-abl and prematurely terminated its translation. The destruction of bcr-abl gene suppressed cell proliferation and induced cell apoptosis in CML lines and in CML stem/progenitor cells. Moreover, the RFNs significantly impaired the leukemogenic capacity of CML cells in xenograft model.

**Conclusion:**

These results illustrate that the RFNs can target to disrupt bcr-abl gene and may provide a new therapeutic option for CML patients affiliated by drug resistance or disease relapse.

**Electronic supplementary material:**

The online version of this article (10.1186/s13046-019-1229-5) contains supplementary material, which is available to authorized users.

## Background

Chronic myeloid leukemia (CML) is a malignant myeloproliferative disorder initiated from hematopoietic stem cells [1]. It is characterized by t(9;22)(q34;q11) reciprocal translocation, which forms a bcr-abl fusion gene [2–4]. This fusion gene encodes a BCR-ABL protein which harbors constitutive tyrosine kinase activity that could activate multiple signaling pathways such as JAK–STAT [5], MEK-ERK [6, 7] and CRKL, contributing to the induction of malignant proliferation and apoptosis inhibition [8]. The tyrosine kinase inhibitors (TKIs) are used for the treatment of CML and have achieved a favorable therapeutic effect [9–12]. However, the rate of TKIs resistance has been over 25% and the treatment effect of TKIs to TKIs resistant or disease relapsed patients is unsatisfactory [13–15].

The bcr-abl fusion gene is the primary cause that leads to the pathogenesis of CML and drug resistance even disease relapse. Theoretically, disruption of bcr-abl fusion gene would disturb its translation into BCR-ABL protein, thus thoroughly settle the problem of CML initiation and drug resistance. Genome editing is a vigoroso means to alter gene function which has been broadly applied to disease therapy. Our team previously tried to use zinc finger nucleases (ZFNs) to edit the bcr-abl gene [16] and Ignacio García-Tuñón et al. adopted monomeric Cas9 to modify Boff-p210 of murine cells [17]. Both these researches have achieved some progresses in CML treatment which means it is feasible to treat CML via editing bcr-abl.

However, the design of ZFNs is heavily dependent on the sequence of DNA which gravely limits the number of target site. Besides, it requires protein engineering of ZFNs if changing targets of interest [18]. Meanwhile, high off-target rate and low specificity of monomeric Cas9 render its unsuitability for human gene editing [19, 20]. These defects limit the application of the two technologies for CML therapy. Therefore, it remains essential to find an alternative strategy for CML patients, particularly for those suffer from TKIs resistance or disease relapse. To solve these problems, the CRISPR RNA-guided FokI nucleases (RFNs) were introduced in our research [21]. We adopted the strategy of fusing FokI with CRISPR/Cas9 to edit the bcr-abl gene, which enabled the universality of CRISPR site design and the specificity of FokI cleavage. It may provide us an alternative to overcome the limitation of current treatment for CML.

The RFNs system consists of a catalytically dead Cas9 (dCas9, D10A, H804A) and the FokI restriction endonuclease cleavage domain [22]. The guide RNA (gRNA) forms a scaffold that binds dCas9 and pairs with the target sequence which determines the specificity of DNA cleavage. The FokI restriction endonuclease is responsible for cleaving DNA at the site bound by dCas9. To achieve successful DNA cleavage, it requires two distinct FokI-dCas9 monomers to simultaneously bind as heterodimer, because the monomeric FokI nuclease domains have no catalytic competence [23]. The dimeric RFNs allow the system to be less tolerant of mismatches and highly increases the specificity for more than 140 times compared with CRISPR/Cas9 [23].

In cells, double strand breaks (DSBs) could be generated by the corresponding gRNA which forms a scaffold that binds FokI-dCas9 system to active. The broken ends of DSBs could be repaired through non-homologous end joining (NHEJ) which might create insertions or deletions (indels) because of nucleotide mismatches [24, 25], or repaired precisely through homology-directed repair (HDR) if provided an exogenous DNA template [26]. The NHEJ repair is unpredictable while the HDR repair is predictable. If the homologous DNA template is provided, the HDR rate will highly increase [27]. Therefore, we designed several pairs of gRNA to guide FokI-dCas9 nuclease to cleave targeted sequence of bcr-abl oncogene. Exogenous DNA template (donor) containing 8-base sequence of *Not*I enzyme digestion site was provided meanwhile to initiate HDR at the DSBs. If the 8-base sequence of *Not*I enzyme digestion site inserts into the bcr-abl sequence by HDR, the reading frame of bcr-abl will shift and a stop codon may generate ahead of time downstream the RFNs cleavage site, then BCR-ABL translation would prematurely terminate. Generally, we designed an approach based on RFNs to edit bcr-abl gene and evaluated whether it could effectively cleave the bcr-abl gene, then down-regulated the expression level of BCR-ABL and relevant signal pathway downstream of it in vitro. Then we detected its effect on the oncogenesis of BCR-ABL in xenograft model.

## Methods

### Cell line and treatment

K562 (ATCC), K562/G01, U937 (SIBCB, China) and HL60 (SIBCB, China) cell lines were cultured in RPMI-1640 (BI, USA) with 10% fetal bovine serum (FBS) supplemented. AD293 (SIBCB, China) cells were grown in Dulbecco’s modified Eagle’s medium (DMEM) that contains 10% FBS (Lonsera, USA). K562/G01 is an imatinib-resistant cell line which obtained from K562 treated with persistently increased concentration of imatinib that up to 5 mg/L for several months [28]. All of these cells were maintained in a humidified atmosphere with 5% CO_2_ at 37 °C.

### Information of clinical samples

Eight bone marrow samples were provided by the first affiliated hospital of Chongqing Medical University. In which, three samples were from individuals diagnosed with leukocytosis or anemia, three cases from primary diagnosed CML patients and two from relapsed CML patients. The specific information of CML patients were shown in Additional file 1: Table S1.

### Isolation of hematopoietic stem cells

Mononuclear cells were separated from bone marrow samples with the human bone marrow mononuclear cell isolation kit (#LTS1077–1, TBD science, China). CD34 positive cells were isolated with Stemsep human CD34 positive selection cocktail (#14756, STEMCELL Technologies, Canada) from separated mononuclear cells and grown in StemSpan serum-free expansion medium (#09655, STEMCELL Technologies, Canada) [16] containing 20 ng/ml IL-3 (#200–03, PeproTech, USA), 50 ng/ml SCF (#300–07, PeproTech, USA) and 20 ng/ml IL-6 ((#200–06, PeproTech, USA)) in 5% CO_2_ at 37 °C. The research conformed to the standard stipulated by Declaration of Helsinki and was performed with the approval of the ethical committee of Chongqing Medical University.

### gRNA design and construction

The gRNA empty vector and FokI-dCas9 expression vector were obtained from Addgene (Addgene plasmid #53370 and #53369). The gRNA sequences were designed in the ZiFiT website (http://zifit.partners.org/ZiFiT/ChoiceMenu.aspx) and each double-site gRNA contains left-oligoduplex and right-oligoduplex (Additional file 1: Table S2). To construct the gRNA expression vector, the complementary oligos for each gRNA that containing 4 bp-overhang sequence were gradiently annealed and ligated with middle oligo, thus these three oligoduplexes were assembled as one oligoduplex. The middle oligo sequences were synthesized as Additional file 1: Table S2. Finally, this assembled oligoduplex was ligated with linearized gRNA empty vector which had been digested with BsmBI (New England Biolabs, UK). The gRNA-half-site (gRNA-half) contains only one oligoduplex, and can simply ligate with linearized gRNA empty vector after denaturation and gradiently anneal. The ligated gRNA was transformed into competent cells, and single colonies were cultured and expanded before plasmid extraction with TIANprep Mini Plasmid Kit (Tiangen, China). The constructed gRNA expression plasmids were verified by Sanger sequencing.

PCR fragments with approximately 35-bp homology arms were reported to function as efficacious donors for genome editing [29], thus we synthesized donor sequence and inserted 8-base sequence of *Not*I digestion site into it. The sequence was shown in Additional file 1: Table S3. The synthesized donor oligos were used as template and amplified by PCR using the primer 5′- CCTTCAGCGGCCAGTAGCATC-3′ and 5′- CTTGGAGTTCCAACGAGCGG-3′.

### Necleofection

Plasmids of FokI-dCas9, gRNAs and Donor were transfected with the cell line nucleofector kit V (#VCA-1003, Lonza Bioscience, Switzerland) or the CD34^+^ cells nucleofector kit (#VPA-1003, Lonza Bioscience, Switzerland) with the use of the Amaxa Nucleofector II device (#AAB-1001, Lonza Bioscience, Switzerland) [16]. Firstly, 1.5 ml of culture medium was added in a 12-well plate and pre-equilibrated in a humidified incubator under the condition of 5% CO_2_ at 37 °C. Secondly, 1 × 10^6^ cells were prepared and centrifuged at 200 × g for 10 min. After centrifugation, the supernatant was removed completely and cells were blended with DNA plasmids in 100 μl pre-mixed nucleofector solution (82 μl of Solution V and 18 μl of supplement I). Thirdly, the cell/DNA suspension was transferred into the nucleofector cuvette and nucleofected using the program U-008 for CD34^+^ cells, T-016 for K562, W-001 for U937, and T-019 for HL60, respectively. Approximately 500 μl of pre-equilibrated culture medium was added into the cuvette. Finally, the cell/DNA suspension was gently transferred into the prepared 12-well plate and cultured in humidified 5% CO_2_ incubator at 37 °C.

### HDR efficiency analysis

To estimate the HDR efficiency, genomic DNA from K562 cells transfected with RFNs (gRNA plus FokI-dCas9) plus donor was extracted after 60 h of treatment and amplified by PCR. The PCR primers were designed at the outside of the double breaks of cut site. The pcr primer was using a forward primer (5′-TATTTTTGCTTCTGAGAATAAAACT-3′) binding to the intron before c-abl exon 2, and a reverse primer (5′-CAAAGGGTGGTAGGTCAAAC-3′) binding to the c-abl exon 2. As the donor contains the *Not*I restriction site, HDR events could be detected with *Not*I restriction enzyme digestion according to the reaction system of *Not*I (TaKaRa, Japan). The digestion product was detected by DNA-PAGE electrophoresis with 8% agarose gel.

### T7E1 assay

Mismatches upon RFNs mediated DSBs induction at the expected genomic locus in K562 cells were evaluated by T7E1 assay. The genomic DNA of K562 cells treated with RFNs was extracted with TIANamp Genomic DNA Kit (Tiangen, China). Target sequence surrounding cleavage site were amplified by PCR. The primer sequences of PCR were 5′-TATTTTTGCTTCTGAGAATAAAACT-3′ and 5′-CAAAGGGTGGTAGGTCAAAC-3′. PCR products of wild type cells and edited cells were mixed and annealed for the sake of forming heteroduplex DNA, then digested with T7E1 at 37 °C for 15 min [30]. The digestion product was detected by DNA-PAGE electrophoresis.

### CCK-8 assay

For CCK-8 assay, 3 × 10^3^ cells of each treated groups were plated in per well of 96-well plate, then cultured in 100 μl RPMI-1640 added 10% FBS in a humidified incubator with 5% CO_2_ at 37 °C. Each well was added with 10 μl CCK-8 (MCE, USA) at indicated time and incubated for 3 h at 37 °C in darkness. Then absorbance value was detected at 450 nm. Each group included four counterparts and was performed for three times.

### Cell Colony-forming assay

Three hundred treated cells suspended in 750 μl medium were plated in per well of 24-well plate, then 750 μl of 2.7% methylcellulose (Sigma, USA) was added in and stirred well with the medium. After culturing for 7–10 days, the number of colonies was counted with an inverted micro-scope (Nikon C-HGF1, Japan). Each group included four counterparts and was repeated for three times.

### Western blot

BCR-ABL protein and relevant signal molecules were assessed by western blotting [1] using rabbit anti-phospho-BCR-ABL (#2868), anti-MEK1/2 (#9122), anti-BCR-ABL (#2862), anti-STAT5(#94205), anti-phospho-STAT5 (#9314), anti-phospho-ERK (#9101), anti-phospho-CRKL (#3181), anti-Caspase-3 (#9662), anti-CRKL (#38710) and anti-PARP (#9532) antibody with 1:1000 dilution (Cell Signaling Technology, USA). Horseradish peroxidase-conjugated α-rabbit antibody (Introvigen, USA) was used as a secondary antibody at 1:5000 dilution. Detection of blot was performed using super ECL plus western blotting substrate (Baoguang, China).

### Immunofluorescence imaging

Cells were harvested, washed and then coated on slides, fixed (30 min, 4% paraformaldehyde solution), permeabilized (15 min, 0.2% Triton X-100), blocked (2 h, 1% BSA) and then incubated with primary antibodies (1:200 in 5% goat serum). After incubation with fluorescent-labeled secondary antibody (Introvigen, USA) in darkness for 1 h at 37 °C, cells were stained with diluted DAPI (1:1000 in PBS).

### Murine xenograft leukemogenesis

Six- to seven-week-old female NOD/SCID mice were adopted for our experiment (5 mice for each group). Mice were received 250 cGy radiation before injection. Leukemia xenografts were induced by intravenous injection with 5 × 10^6^ treated K562/G01 cells suspended in 0.2 ml PBS. White blood cells (WBCs) were counted and body weight of mice was monitored every week. In order to measure the percentage of human CD45^+^ cells, cells from peripheral blood was collected, incubated with anti-CD45 antibody, and then measured by flow cytometry. The NOD/SCID mice were purchased from Beijing HFK Bioscience Co. Ltd. (China) which obtained the laboratory animal production license. The use of NOD/SCID mice and the animal experiments were approved by the Ethics Committee of Chongqing Medical University.

### Statistical methods

All statistical results were shown as mean ± SD. The statistical significance among each group was assessed by one-way ANOVA analysis. Statistical analyses were calculated with GraphPad Prism 5.0 software. Results with statistically significance were marked with asterisks. *P* < 0.05 was considered to reach statistical significance.

## Results

### The design of RNA guided-FokI nucleases

**Fig. 1:**
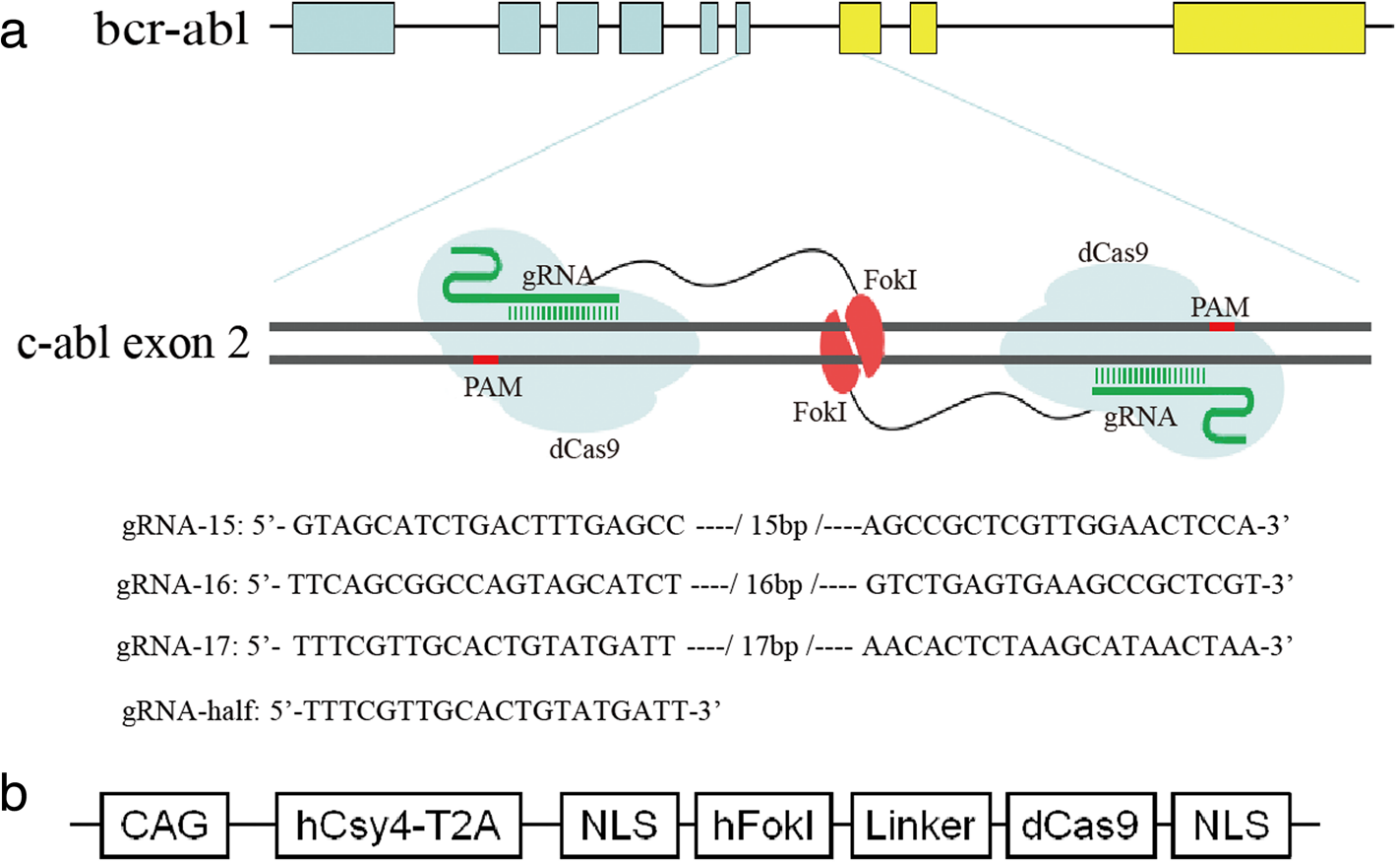
Schematic diagram of dimeric RFNs target bcr-abl to induce gene modification. **a** Dimeric FokI-dCas9 proteins target to DNA half-site sequences with PAM out orientation by pairs of gRNAs to cleave the intervening spacer sequence of c-abl exon 2. The gRNA-15, gRNA-16, gRNA-17, and gRNA-half showed the target site in c-abl exon 2. **b** The structure of FokI-dCas9 nucleases expression plasmid

As most kinase domain mutations occur in the abl part of bcr-abl oncogene [31], we intended to disrupt the c-abl exon 2, which is the nearest exon of c-abl to bcr-abl fusion site. As shown in Fig. 1a, gRNA recruited FokI-dCas9 proteins target to DNA half-site sequences with protospacer adjacent motif (PAM) out orientation to cleave the intervening spacer sequence of c-abl exon 2. The PAM is essential to nuclease activation. For the FokI-dCas9 nucleases, it was co-expressed with Csy4 and initiated by the CAG promoter, separated with Csy4 by a self-cleaving peptide named T2A, and flanked by nuclear localization signal (NLS) sequence [21, 32]. A five-amino-acid sequence in form of GGGGS functioned as a linker that ligated the FokI nuclease and dCas9 domains (Fig. 1b) [21].

### The bcr-abl oncogene in CML cell line was efficiently and specifically disrupted by RFNs

**Fig. 2:**
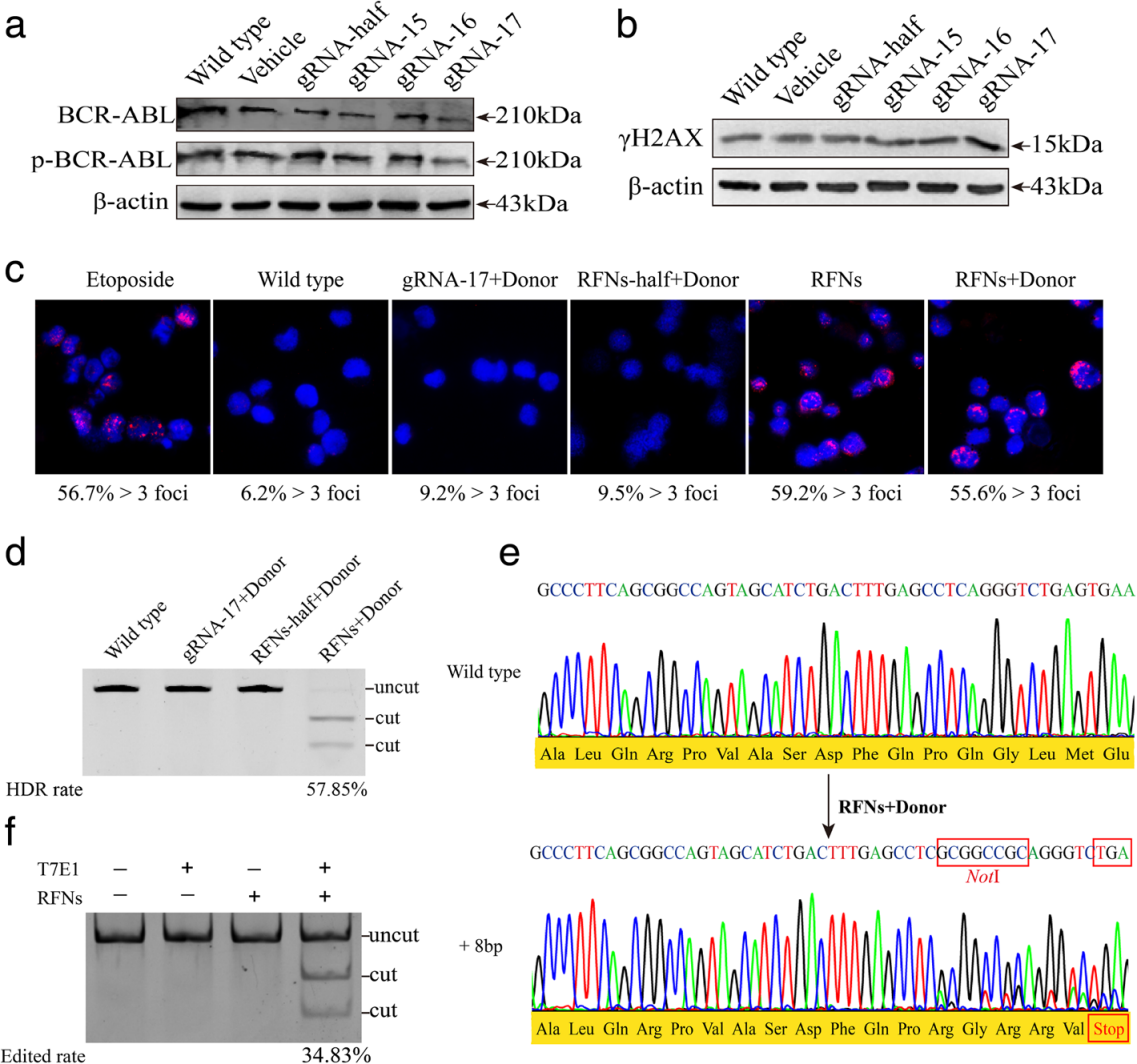
RFNs induce gene modification of bcr-abl. **a** Detection of BCR-ABL expression by western blot. Vehicle or each gRNA combined FokI-dCas9 plus donor were co-transfected into K562 cells. **b** Examination of the amount of γH2AX by western blot. K562 cells were transfected with vehicle or each gRNA guided FokI-dCas9 plus donor. **c** DSBs induced by RFNs were detected with 53BP1 immunostaining. Etoposide treated K562 cells were adopted as positive control and wild type K562 cells were used as negative control. Different groups of plasmids transfected K562 cells were harvested after 60 h of treatment. Foci that form more than 3 in cells were considered to be 53BP1 positive and the positive rates were shown beneath each panel. **d** The HDR rate of RFNs plus donor on bcr-abl was detected by *Not*I digestion. The “uncut” indicated the location of pcr fragment of wild type and “cut” showed the location of digested fragment by *Not*I restriction enzyme. **e** Predicted HDR was verified by Sanger sequencing. The sequence of RFNs plus donor treated cells was analyzed with in silico analysis. Result showed the *Not*I sequence was inserted into bcr-abl and a TGA stop codon was generated downstream of the cleavage site. **f** The edited rate by RFNs was detected via T7E1 assay. The “cut” bands indicated occur of “indels”

To assess human gene editing with RFNs and determine whether RFNs could introduce DSBs at bcr-abl, we designed three pairs of gRNA and one half-site gRNA (gRNA-half) with ZiFiT Targeter Vesion 4.2 software. gRNA expression plasmids were constructed and delivered into K562 cell line with FokI-dCas9 and donor. As the result of western blot shown in Fig. 2a, we found that when gRNA-17, FokI-dCas9 and donor were co-delivered into K562 cells, the expression level of phosphorylated BCR-ABL and BCR-ABL significantly decreased. gRNA-15, gRNA-16 and gRNA-half guided FokI-dCas9 groups had weak effect on the expression of phosphorylated BCR-ABL and BCR-ABL (Additional file 2: Figure S1a). This result indicated that the target site guided by gRNA-17 was effective for bcr-abl editing. The phosphorylated histone H2AX (γH2AX) is a DNA damage marker [33, 34]. If gRNA guided FokI-dCas9 effectively cleaved bcr-abl gene, DSBs would generate and the γH2AX would increase. The western blot result displayed that the expression of γH2AX distinctly increased in K562 cells co-transfected with RFNs and donor, especially in gRNA-17 guided FokI-dCas9 group. There was no significant difference in vehicle or gRNA-half guided FokI-dCas9 group (RFNs-half+Donor) compared with wild type group (Fig. 2b, Additional file 2: Figure S1b). This result illustrated that RFNs can induce DSBs in K562 cells. Hence we chose the gRNA-17 as the main gRNA for subsequent research. As previously reported that p53-binding protein 1 (53BP1) could be recruited to DSB sites in early time and form foci at DNA cleavage sites [35]. We detected 53BP1 with immunofluorescence to measure the formation of DSBs. Etoposide is a chemotherapy medication that could cause DNA damage. Thus etoposide group is set as positive control. As shown in Fig. 2c, etoposide treated cells had high level of 53BP1 (51.5% > 3 foci). Background level of 53BP1 was observed in untreated K562 cells or K562 cells co-delivered with gRNA-17 plus donor, even in K562 cells treated with RFNs-half plus donor (6.2% > 3 foci, 9.2% > 3 and 9.5% > 3 foci respectively). In contrast, high level expression of 53BP1 foci was observed in K562 cells treated by RFNs with donor or not (55.6% > 3 foci and 59.2% > 3 foci respectively). These results showed that RFNs could effectively induce DSBs in K562 cells.

To investigate whether the RFNs and donor could induce termination of BCR-ABL translation, gRNA-17 plus donor, RFNs-half plus donor, and RFNs plus donor were co-delivered into K562 cells. 60 h later, genomic DNAs were extracted and PCR amplified, then digested by *Not*I restriction enzyme. HDR efficiency of bcr-abl was 57.85% (Fig. 2d). The result of Sanger sequencing showed that the 8-base sequence of *Not*I was successfully inserted into bcr-abl. Moreover, the frame shift mutation of bcr-abl induced by *Not*I sequence insertion produced a premature stop codon at the downstream of cut site and fundamentally terminated the bcr-abl translation (Fig. 2e).

DSBs introduced by RFNs are primarily repaired by NHEJ, which often generates “indels” around cleaving site. If indels emerged and formed mismatches with wild type DNA, it could be detected via T7E1 assay because T7E1 enzyme is sensitive to DNA mismatches. The results exhibited that indels did exist in RFNs edited K562 cells. The modification rate of bcr-abl gene reached 34.83% (Fig. 2f). In contrast, there was no indels detected in wild type group, gRNA-17 plus donor group or RFN-half plus donor group. These results revealed that the RFNs designed in our experiments could targeted edit bcr-abl with high efficiency.

To predict the potential off-target sites of RFNs with the target site of gRNA-17, we blast the target sequence of gRNA-17 in form of “CCNTTTCGTTGCACTGTATGATTNNNNNNNNNNNNNNNNNAACACTCTAAGCATAACTAANGG” with human genome sequence from UCSC (hg19) in the website of (https://bitbucket.org/vishalthapar/casper-scan) which is designed for predicting potential off-target sites of RFNs. The number of allowed mismatches was set as 12 and it predicted 1 potential off-target site (Additional file 1: Table S4). However, we did not detect any off-target with deep sequencing. This result revealed that the RFNs we designed could target bcr-abl with high specificity.

### The expressions of BCR-ABL oncoprotein and its downstream signaling molecules were decreased in RFNs treated CML cells

**Fig. 3:**
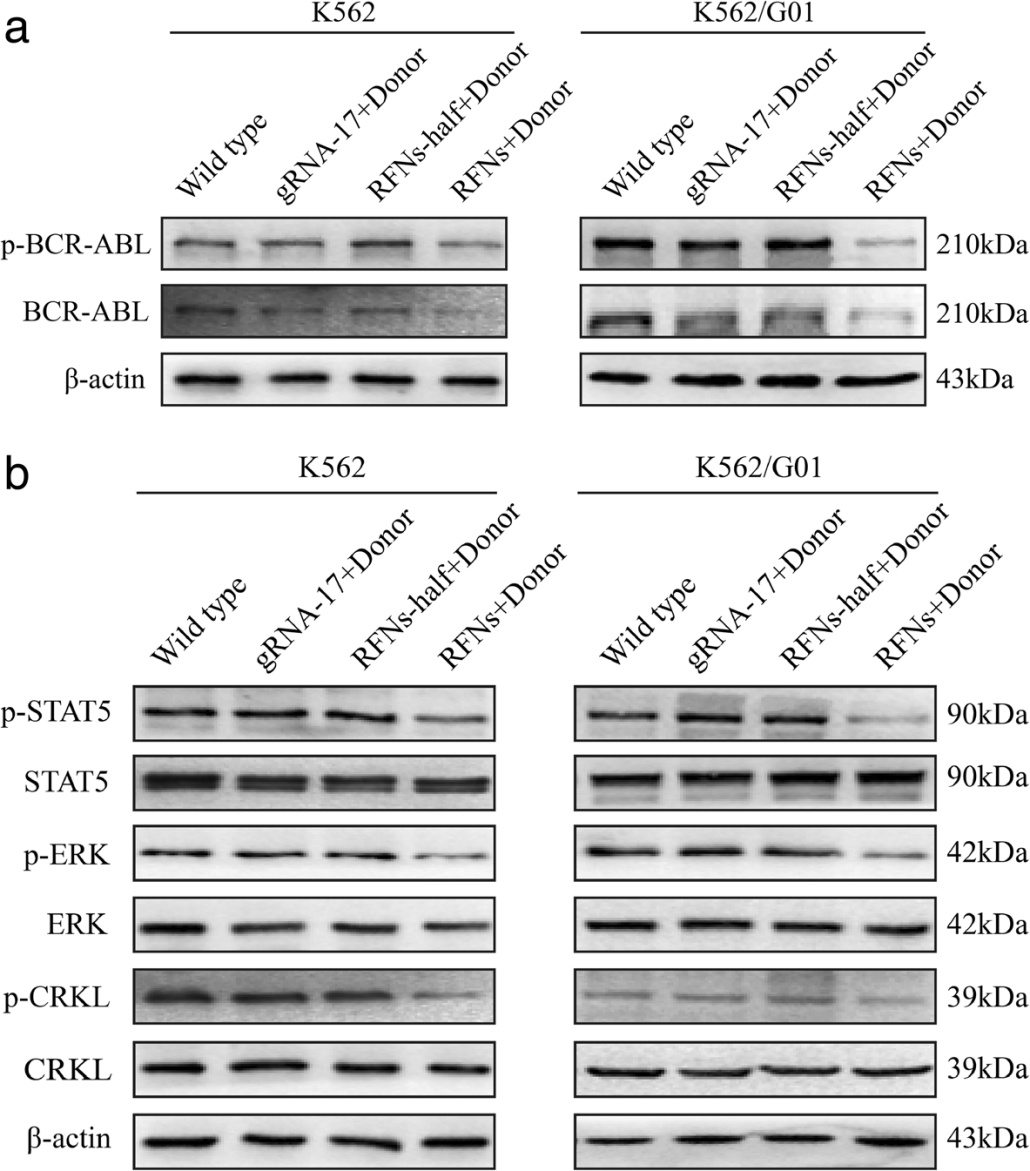
The expressions of BCR-ABL oncoprotein and its downstream signaling molecules were decreased in RFNs treated CML cells. K562 and K562/G01 cells were co-delivered with gRNA-17 plus donor, RFNs-half plus donor, RFNs plus donor, respectively. Proteins were extracted after 60 h of transfection and analyzed by western blot. Expressions of p-BCR-ABL and BCR-ABL were reduced in RFNs plus donor group (**a**). The activated phospho-CRKL, phospho-ERK and phospho-STAT5 were down-regulated in RFNs plus donor group (**b**)

To investigate whether RFNs system could prevent BCR-ABL translation, we performed western blot assay to evaluate the expression level of BCR-ABL. The result revealed that no matter in K562 cells or in K562/G01 cells, the expression levels of BCR-ABL and its phosphorylation were significantly decreased in the RFNs plus donor group compared with those in the groups of wild type, gRNA-17 plus donor, and RFNs-half plus donor (Fig. 3a). There was still trace amount of BCR-ABL protein detected in RFNs plus donor group, since the protein was extracted from mixed cells which were limited by the RFNs modification rate and transfection efficiency. As FLAG tagged at the C-terminal of FokI-dCas9, the transfection efficiency of K562 cells could be estimated by detecting the percentage of FLAG positive cells by immunofluorescent assay (Additional file 3: Figure S2a). The statistical analysis of immunofluorescent assay showed that the transfection efficiency of K562 cells was 87.3% (Additional file 3: Figure S2b). Next, we measured the main signaling molecules downstream of BCR-ABL. We noticed that the expression level of phospho-CRKL, phospho-ERK and phospho-STAT5 down-regulated significantly (Fig. 3b). In general, these results suggested the expression level of BCR-ABL protein and the activation of signaling molecules downstream of BCR-ABL could be down-regulated by RFNs system.

### The proliferation was suppressed and apoptosis was induced by RFNs in imatinib sensitive and resistant CML cells

**Fig. 4:**
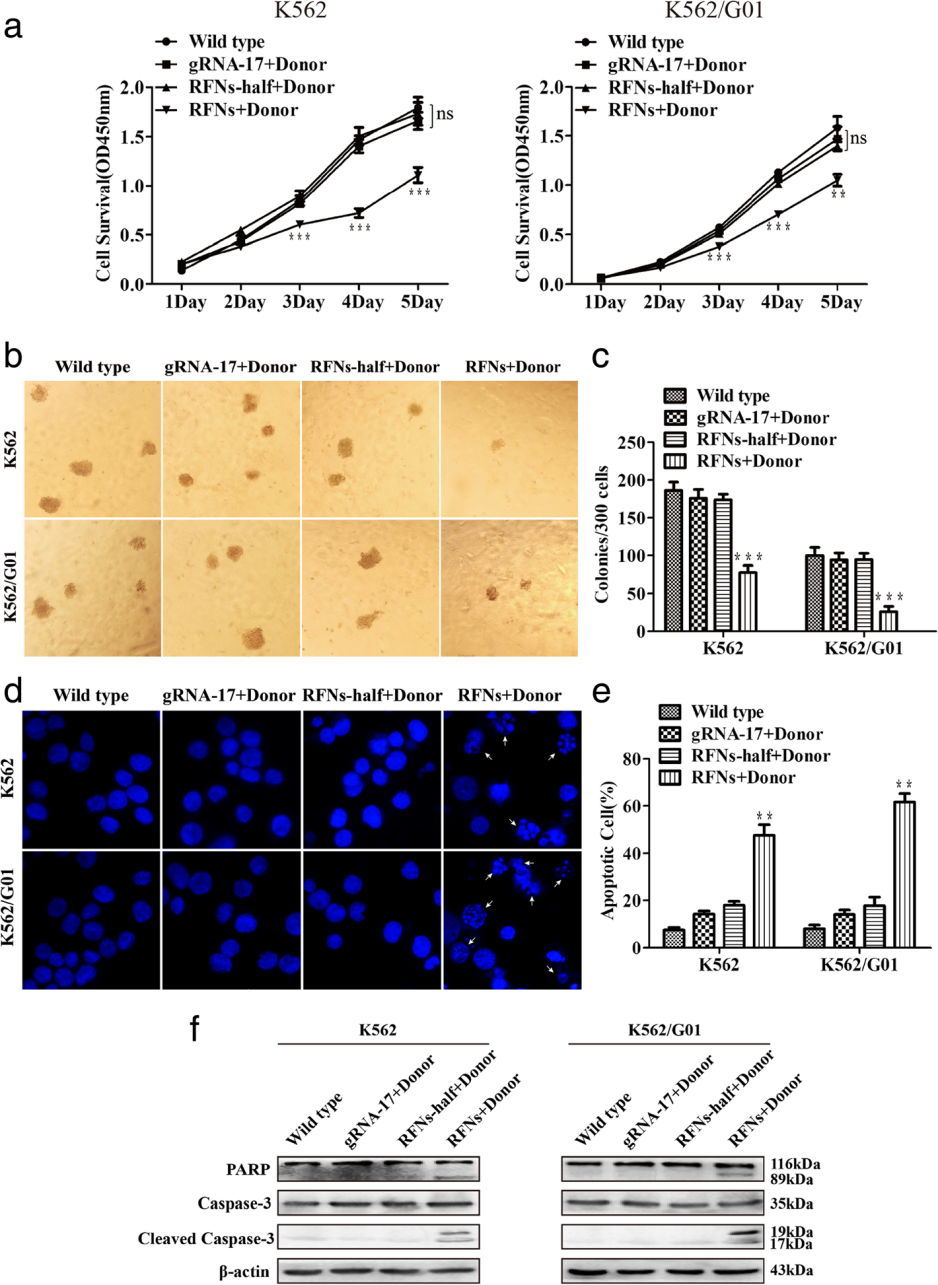
RFNs suppress viability and induce apoptosis of imatinib sensitive and resistant cells. Cells were transfected with gRNA-17 plus donor, RFNs-half plus donor, RFNs plus donor, respectively. **a** Evaluation of cell proliferative capacity by CCK-8 assay. **b**, **c** The capacity of colony formation was assessed by colony-forming assay. **d** Cell nucleuses were stained by DAPI to detect the morphologic changes caused by apoptosis. The white arrows pointed out the typical apoptotic cells. **e** Satistical analysis result of apoptotic cells tested by flow cytometry. **f** The activation of apoptotic pathway was investigated by western blot. The results are presented as the means ± SD. One-way ANOVA analysis was used to compare treated groups with control group, *p* < 0.01 (**) and *p* < 0.001 (***)

To test the effect of RFNs system on bcr-abl editing, we transfected the imatinib sensitive and resistant cells with RFNs plus donor. Then colony-forming assay and CCK-8 assay were carried out to detect the viability of cells. As displayed in Fig. 4a, we noticed that cell survival ability of RFNs plus donor group was significantly lower than other groups. Besides, there were no obvious differences of viability among other groups. It suggested that the cell proliferation of CML was suppressed by RFNs. The result of colony-forming assay also showed similar results (Fig. 4b, c).

Parrallelly, we performed DAPI staining to determine whether the apoptosis of CML cells could be induced by RFNs. DAPI staining was performed to observe nuclear morphology. The result showed that the amount of cells with nuclear morphology changes such as karyopyknosis and karyorrhexis obviously increased in RFNs plus donor group. There were no remarkable nuclear changes observed in other groups (Fig. 4d). To verify this phenomenon, apoptosis was measured by flow cytometry. Similarly, we observed that the apoptosis rate in the group treated with RFNs plus donor was dramatically higher than those in other groups (Fig. 4e and Additional file 4: Figure S3a). Furthermore, to confirm which apoptotic pathway was activated, we carried out western blot assay and found that the expression of activated PARP and caspase-3 increased. These data manifested that the caspase pathway was activated both in imatinib sensitive and resistant CML cells treated with RFNs plus donor (Fig. 4f).

Furthermore, to test whether the RFNs system might cause cytotoxic effect on bcr-abl negative cells, we performed same treatment in U937, HL60 and AD293 cells. The result of CCK-8 assay showed that the proliferation had no significant change among all groups, which meant the RFNs system barely had cytotoxic effect on bcr-abl negative cells (Additional file 4: Figure S3b). Taken together, these results revealed that the RFNs inhibited proliferation and induced apoptosis of imatinib sensitive and resistant CML cells.

### The proliferation was suppressed and apoptosis was induced by RFNs in CML stem/progenitor cells

**Fig. 5:**
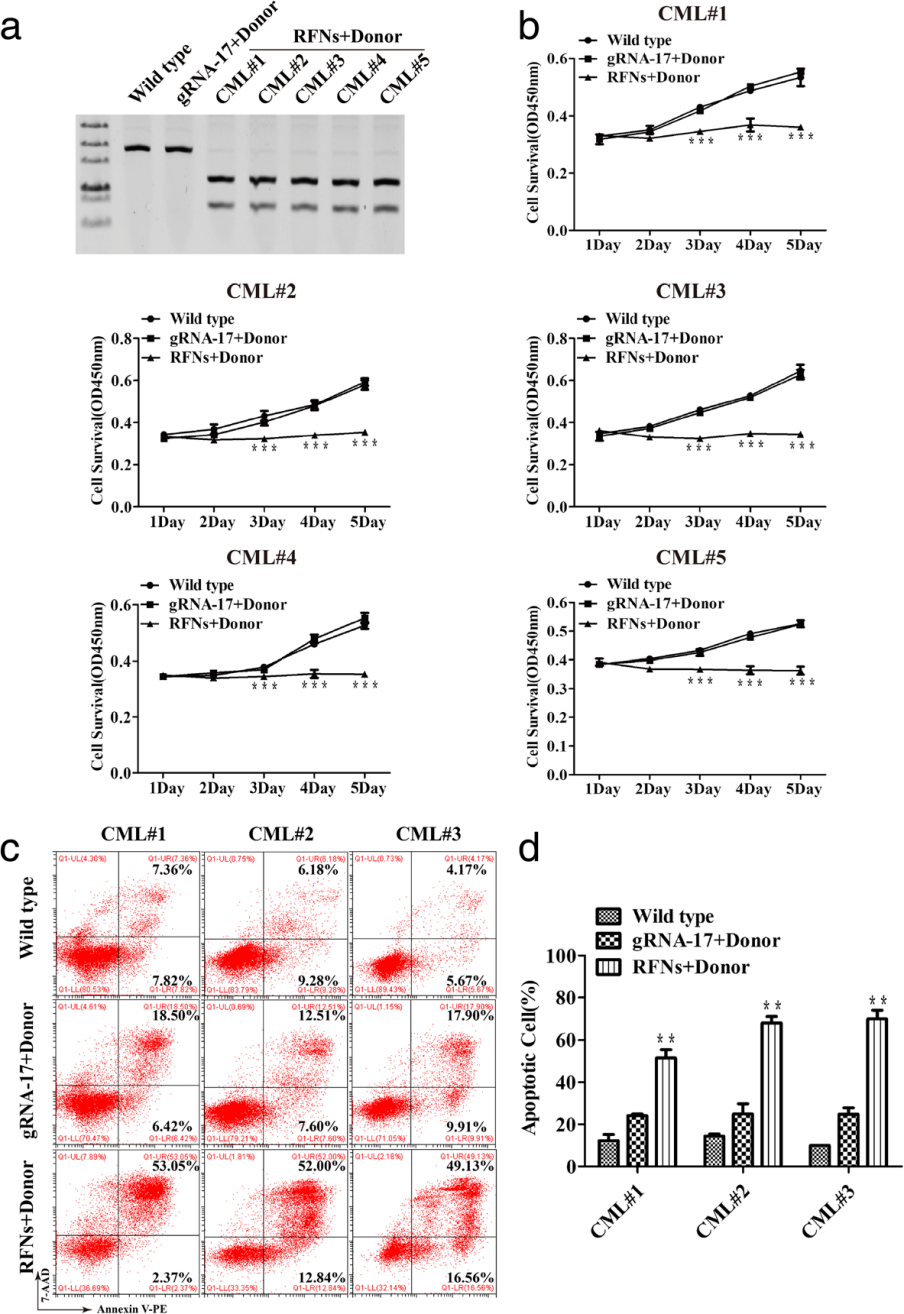
The proliferation was suppressed and apoptosis was induced by RFNs in CML stem/progenitor cells. CML CD34^+^ cells were transfected with gRNA-17 plus donor, and RFNs plus donor. **a** The evaluation of modification efficiency of RFNs on bcr-abl by *Not*I digestion. **b** Cell viability of CML stem/progenitor cells was tested by CCK-8 assay. **c**, **d** Percentage of apoptotic CML stem/progenitor cells was tested by flow cytometry. All results are exhibited as the mean ± SD, p < 0 .01 (**) and p < 0.001 (***)

The existence of CML stem/progenitor cells is an important reason for drug resistance or disease relapse due to their lack of sensitivity to TKIs and poor absorption rate of TKIs. In order to confirm if the RFNs system was effective on CML stem/progenitor cells, the CML CD34^+^ cells were co-delivered with RFNs plus donor. The genomic DNA of CML CD34^+^ cells were prepared and PCR amplified. HDR events were detected by *Not*I restriction enzyme digestion. The digestion result showed that the PCR fragment of genomic DNA from RFNs plus donor treated CML CD34^+^ cells could be digested by *Not*I restriction enzyme (Fig. 5a). Then, we tested the proliferative capacity of treated cells via CCK-8 assay and the apoptotic rate by flow cytometry. The data of CCK-8 assay displayed that the RFNs plus donor could remarkably inhibit the proliferative capacity of CML stem/progenitor cells (Fig. 5b). The result of flow cytometry displayed that the amount of apoptotic cells dramatically increased in the RFNs plus donor treated group, indicating that the RFNs promoted apoptosis of CML primary stem/progenitor cells (Fig. 5c, d). What’s more, we tested the effect of RFNs on bcr-abl negative CD34^+^ cells from leukocytosis or anemia individuals, and found that RFNs did not influence the viability and apoptosis of these cells (Additional file 5: Figure S4a-d).

### The leukemogenic capacity of bcr-abl in mice was impaired by RFNs

**Fig. 6:**
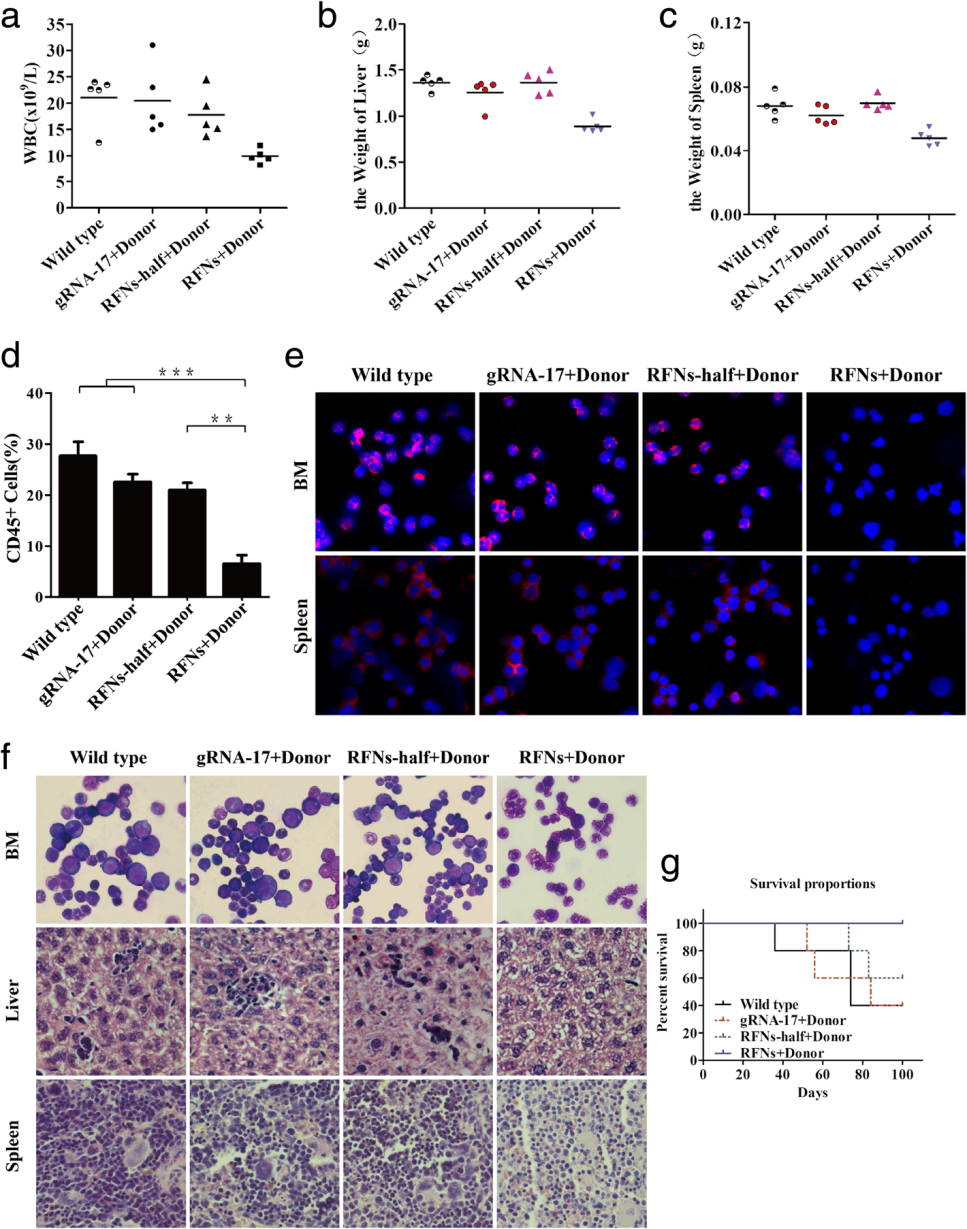
The leukemogenic capacity of bcr-abl in mice was impaired by RFNs. **a** WBC counts in each group were counted. The maximum value of each mouse was recorded. **b**, **c** The weights of spleen and liver of mice in each group were measured. **d** The amount of human CD45^+^ cells was tested by flow cytometry. **e** The expression of BCR-ABL protein in each group was detected by immunofluorescent assay. **f** Immature cells from bone marrow were checked by Wright’s stain and the infiltration of spleen and liver was analyzed by H&E stain. **g** Kaplan-Meier method to analyze the survival curves of mice in each group

To detect whether disruption of bcr-abl could impair its pathogenicity in vivo, the NOD/SCID mice receiving sublethal dose of radiation were intravenously injected with wild type K562/G01 cells or K562/G01 cells co-transfected with gRNA-17 plus donor, RFNs-half plus donor, RFNs plus donor, respectively. As shown in Fig. 6a, WBC counts of the mice from RFNs plus donor group were lower than those of mice from wild type group, gRNA-17 plus donor group, and RFNs-half plus donor group. Spleen and liver of mice were excised and weighted. We observed that mice in the RFNs plus donor group developed mild splenomegaly and hepatomegaly compared with wild type group, gRNA-17 plus donor group, and RFNs-half plus donor group (Fig. 6b, c, Additional file 6: Figure S5a). Solid tumors appeared in some mice of wild type group and gRNA-17 plus donor group (Additional file 6: Figure S5b). To confirm that the leukocytes were derived from human leukemic cells, peripheral blood from each group was collected and the percentage of positive human-CD45 antigen was measured by flow cytometry. The percentage of CD45^+^ cells is used to evaluate the progression of leukemia. We observed that CD45^+^ cells dramatically reduced in RFNs plus donor group (Fig. 6d). Infiltration in liver, spleen and bone marrow was detected by hematoxylin/eosin (HE) staining, Wright’s staining and immunofluorescent assay. The data of immunofluorescent assay exhibited that lower level of BCR-ABL expression was observed in the spleen and bone marrow from RFNs plus donor group compared with wild type group, gRNA-17 plus donor group, and RFNs-half plus donor group (Fig. 6e). In addition, the result of HE staining showed that compared to wild type group, gRNA-17 plus donor group, and RFNs-half plus donor group, less leukemic cell infiltration was observed in liver and spleen from RFNs plus donor group. It showed that the myeloid: erythroid ratio in RFNs plus donor group reduced by Wright’s staining (Fig. 6f). Meanwhile, the data of Kaplan-Meier survival analysis displayed that mice in RFNs plus donor group survived a longer time (Fig. 6g). In summary, these results confirmed that RFNs depressed the proliferative capacity of bcr-abl positive cells and impair their leukemogenic capacity in vivo.

## Discussion

It is well known that CML is a myeloproliferative neoplasm derived from hematopoietic stem cell [36]. The bcr-abl fusion gene produced by t(9;22)(q34;q11) reciprocal translocation plays a crucial part in CML leukemogenesis [2, 37]. It encodes BCR-ABL oncoprotein which harbors persistent tyrosine kinase activity and activates multiple signaling molecules [38–40]. Although the TKIs have achieved decent effect in CML treatment, this therapeutic method is not jack of all trades [41]. Approximately 25% of CML patients still suffer treatment failure due to drug resistance or disease relapse [13–15]. Here, we provide an optional strategy to disrupt bcr-abl gene and eradicate its pathogenicity to cure CML.

The RFNs system is an evolutional version based on CRISPR/Cas9. FokI-dCas9 cleaves DNA only when a heterodimer forms. It stringently depends on the presence of paired gRNA and the existence of PAM, which highly improve its specificity and reduce the off-target effect of RFNs. As directed by paired gRNA composed of left and right half-site, the RFNs is expected to specify the target sequence up to 44 bps [21]. Sequences with this length are quite unique. Although the number of mismatches was set up to 12, we only identified one potential off-target site. RFNs system retains the merits of CRISPR/Cas9 capable of designing multiple target sites. It is easy to retarget new DNA sequence only if changing the sequence of gRNA that complementary with target DNA [18]. In our study, we designed several pairs of gRNA with different spacers and then chose the most efficient pair of gRNA to target bcr-abl and recruit dimeric FokI-dCas9 to cleave bcr-abl. We studied the ability of the RFNs system to disrupt bcr-abl oncogene and its effect on imatinib sensitive and resistant CML cell lines and stem/progenitor cells in vitro, then explored its effect in xenograft CML model.

There are two unique mechanisms—NHEJ and HDR—that involved in DNA repair stimulated by DSBs [42]. As an error-prone repair pathway, the NHEJ occurs during the whole cell cycle and could generate indels, but these kinds of indels are unpredictable. HDR occurs only during S and G2 phase which is less frequent than NHEJ [42]. However, if exogenous DNA template provided, HDR rate could be highly increased at DSBs [43, 44]. In addition, the RFNs gain the advantage of FokI cleavage. Previous article reported the low ligation rate of HDR by Cas9 [45] because it generates blunt cuts whereas the FokI produces sticky ends [18] to achieve high ligation rate. It means RFNs increase the HDR rate as well. Considered these facts, RFNs combined donor has been adopted in our study to achieve predictable indels in bcr-abl gene. In our experiments, we verified our predictable indels that 8-base sequence of *Not*I did integrate in bcr-abl, which caused the frame shift mutation of bcr-abl and produced a premature stop codon at the downstream of break point. As a result, the BCR-ABL translation was terminated.

Actually, we conducted limited dilution methods to isolate and culture single cell colonies. Intriguingly, we observed that there were certain morphological changes in some single cell colonies, such as cell swelling and vacuolation, which were similar to the morphological changes that occurred in cells treated with calicheamicin-γ [46]. We speculated this phenomenon might be related to cell apoptosis. However, these single cell colonies grew slowly and it was difficult to collect enough cells for subsequent studies. That’s why we chose mixed cells in our experiments.

The RFNs showed a strong editing capacity in our research. It can be widely applied in mammalian cells to achieve an efficacious editing proportion with absolutely low off-target rate. Nevertheless, it remains to be improved. On the one hand, the dCas9 protein owns a large size which in a certain degree limits its application because it is hard to package if using virus to transduce cells. As the FokI nucleases function as cutter instead of dCas9, the dCas9 could be replaced by another Cas9 with smaller size such as CjCas9 [47] if it is catalytically inactived. On the other hand, fluorescent tag should be engineered into the RFNs for more intuitively observation. We believe these improvements would broaden its application in gene editing, and it is worthy to be explored.

In summary, we have shown that the RFNs can function as heterodimer to efficiently edit bcr-abl with high specificity and flexibility. Despite many therapeutic strategies were explored for the treatment of CML patients in recent decades, the challenge of eliminating CML remains due to the emergence of TKIs resistance and disease relapse. This is the first time RFNs has been introduced in genome editing in CML. We believe it would be a promising therapeutic option for CML patients with TKIs resistance or disease relapse.

## Conclusions

In this study, we have shown that the RFNs plus donor is able to efficiently modify bcr-abl and truncate BCR-ABL oncoprotein. The combination of RFNs and donor could inhibit viability and induce apoptosis of CML cells in vitro. In addition, it could impair the leukemogenic capacity of CML cells in vivo. This approach provides a novel therapeutic option for CML patients affiliated by TKIs resistance or disease relapse.

## Additional files

Additional file 1: **Table S1.** Patients’ information. **Table S2.** The Oligos sequences designed for each gRNA. **Table S3.** Information of Donor. **Table S4.** Potential off-target sites of RFNs. (DOCX 20 kb)

Additional file 2: **Figure S1.** Expressions of relative proteins were detected by western blot. K562 cells were treated with vehicle plasmids, RFNs-half plus donor, RFNs-15 plus donor (gRNA-15), RFNs-16 plus donor (gRNA-16), and RFNs-17 plus donor (gRNA-17), respectively. Non-transfected K562 cells were considered as wild type group. (a) The relative expressions of p-BCR-ABL and BCR-ABL normalized to β-actin were quantified. (b) The relative expressions of γH2AX normalized to β-actin were quantified. The results are presented as the means ± SD. *p* < 0 .01 (**) and *p* < 0.001 (***). (TIF 264 kb)

Additional file 3: **Figure S2.** The transfection efficiency of K562 cells was estimated by detection of FLAG tagged of FokI-dCas9. K562 cells were transfected with gRNA-17 and RFNs respectively. The untreated K562 cells were considered as negative control. (a) FLAG tag was detected by immunofluorescent assay after 48 h of transfection. (b) The percentage of FLAG positive cells was quantified by counting 300 cells in total. (TIF 1277 kb)

Additional file 4: **Figure S3.** (a) RFNs suppress viability and induce apoptosis of imatinib sensitive and resistant cells. Cells were transfected with gRNA-17 plus donor, RFNs-half plus donor, RFNs plus donor, respectively. The apoptotic rate of cells was analyzed by flow cytometry. (b) Cell viability of bcr-abl negative cells was tested via CCK-8 assay. U937, HL60, and AD293 cells were transfected with gRNA-17 plus donor or RFNs plus donor. (TIF 1306 kb)

Additional file 5: **Figure S4.** RFNs have hardly any effect on the proliferation and apoptosis of bcr-abl negative CD34^+^ cells. The bcr-abl negative CD34^+^ cells were isolated from individuals diagnosed with leukocytosis or anemia, and transfected with gRNA-17 plus donor or RFNs plus donor. (a)-(c) Cell viability of normal CD34^+^ cells was detected by CCK-8 assay. (d) Apoptotic proportion of normal CD34^+^ cells was determined by flow cytometry. (TIF 413 kb)

Additional file 6: **Figure S5.** (a) Typical pictures of spleen and liver from wild type group, gRNA-17 plus donor group, RFNs-half plus donor group and RFNs plus donor group. (b) Picture of part solid tumors from wild type group and gRNA-17 plus donor group. (TIF 2127 kb)

## Abbreviations

Chr: chromosome
CML: Chronic myeloid leukemia
DSBs: Double strand breaks
FBS: Fetal bovine serum
HDR: Homology-directed repair
HE: Hematoxylin/eosin
indels: Insertions or deletions
NHEJ: Non-homologous end joining
NLS: Nuclear localization signal
RFNs: RNA-guided FokI nucleases
RFNs-half: RNA-half-site guided FokI nucleases
T7E1: T7 endonuclease 1
TKIs: Tyrosine kinase inhibitors
WBC: White blood cells
