# Supplementary material for: Efficient disruption of bcr-abl gene by CRISPR RNA-guided FokI nucleases depresses the oncogenesis of chronic myeloid leukemia cells
Source: J Exp Clin Cancer Res. 2019 May 28;38:224. doi: 10.1186/s13046-019-1229-5 (PMC6537404; doi:10.1186/s13046-019-1229-5)
Supplement: Supplementary file 1 — Table S1. Patients’ information. Table S2. The Oligos sequences designed for each gRNA. Table S3. Information of Donor. Table S4. Potential off-target sites of RFNs. (DOCX 20 kb) [file 13046_2019_1229_MOESM1_ESM.docx]

**Additional file 1:**

**Table S1.** Patients’ information

| Samples | Diagnosis | Gender | Age, year old | Karyotype | WBC,×10^9^ /L |
| --- | --- | --- | --- | --- | --- |
| CML#1 | Primary CML | female | 53 | t (9;22)(q34.1;q11.2) | 208.23 |
| CML#2 | Primary CML | male | 65 | t (9;22)(q34.1;q11.2) | 243.52 |
| CML#3 | Primary CML | male | 61 | t (9;22)(q34.1;q11.2) | 214.25 |
| CML#4 | Relapse CML | female | 41 | t (9;22)(q34.1;q11.2) | 31.29 |
| CML#5 | Relapse CML | male | 49 | t (9;22)(q34.1;q11.2) | 29.93 |
| Normal#1 | Leukocytosis | male | 32 | normal | 108.23 |
| Normal#2 | Leukocytosis | male | 36 | normal | 11.08 |
| Normal#3 | Anemia | female | 15 | normal | 22.99 |

**Table S2.** The Oligos sequences designed for each gRNA

|  |  | Left-oligoduplex | Right-oligoduplex |
| --- | --- | --- | --- |
| gRNA-15 | Forward | GCAGGGCTCAAAGTCAGATGCTACGTTTTAG | GGCAGAGCCGCTCGTTGGAACTCCA |
|  | Reverse | AGCTCTAAAACGTAGCATCTGACTTTGAGCC | AAACTGGAGTTCCAACGAGCGGCTC |
| gRNA-16 | Forward | GCAGAGATGCTACTGGCCGCTGAAGTTTTAG | GGCAGGTCTGAGTGAAGCCGCTCGT |
|  | Reverse | AGCTCTAAAACTTCAGCGGCCAGTAGCATCT | AAACACGAGCGGCTTCACTCAGACC |
| gRNA-17 | Forward | GCAGAATCATACAGTGCAACGAAAGTTTTAG | GGCAGAACACTCTAAGCATAACTAA |
|  | Reverse | AGCTCTAAAACTTTCGTTGCACTGTATGATT | AAACTTAGTTATGCTTAGAGTGTTC |
| gRNA-half | Forward | GCAGAATCATACAGTGCAACGAAA | |
|  | Reverse | AAACTTTCGTTGCACTGTATGATT | |
| middle oligo | Forward | 5’-pAGCTAGAAATAGCAAGTTAAAATAAGGCTAGTCCGTTATCAACTTGAAAAAGTGGCACCGAGTCGGTGCGTTCACTGCCGTATA-3’ | |
|  | Reverse | 5’-pTGCCTATACGGCAGTGAACGCACCGACTCGGTGCCACTTTTTCAAGTTGATAACGGACTAGCCTTATTTTAACTTGCTATTTCT-3’ | |

**Table S3.** Information of Donor

| **Sequence of Donor** | |
| --- | --- |
| **Forward** | 5’-CCTTCAGCGGCCAGTAGCATCTGACTTTGAGCCTCGCGGCCGCAGGGTCTGAGTGAAGCCGCTCGTTGGAACTCCAAG-3’ |
| **Reverse** | 5’-CTTGGAGTTCCAACGAGCGGCTTCACTCAGACCCTGCGGCCGCGAGGCTCAAAGTCAGATGCTACTGGCCGCTGAAGG-3’ |

**Table S4.** Potential off-target sites of RFNs­

| **Chr** | **Position** | **Strand** | **Mismatch** | **Off-target sequence**  **(LEFT spacer RIGHT )** |
| --- | --- | --- | --- | --- |
| 1 | 179100444 | - | 6 | CCTTTAGTGATGCTGAGTGTGTT  ATCACCACTTGCTACAA  AATCATAAAGTGCAACGAAGAGA |
