# Supplementary figures and images for: Efficient disruption of bcr-abl gene by CRISPR RNA-guided FokI nucleases depresses the oncogenesis of chronic myeloid leukemia cells
Source: J Exp Clin Cancer Res. 2019 May 28;38:224. doi: 10.1186/s13046-019-1229-5 (PMC6537404; doi:10.1186/s13046-019-1229-5)

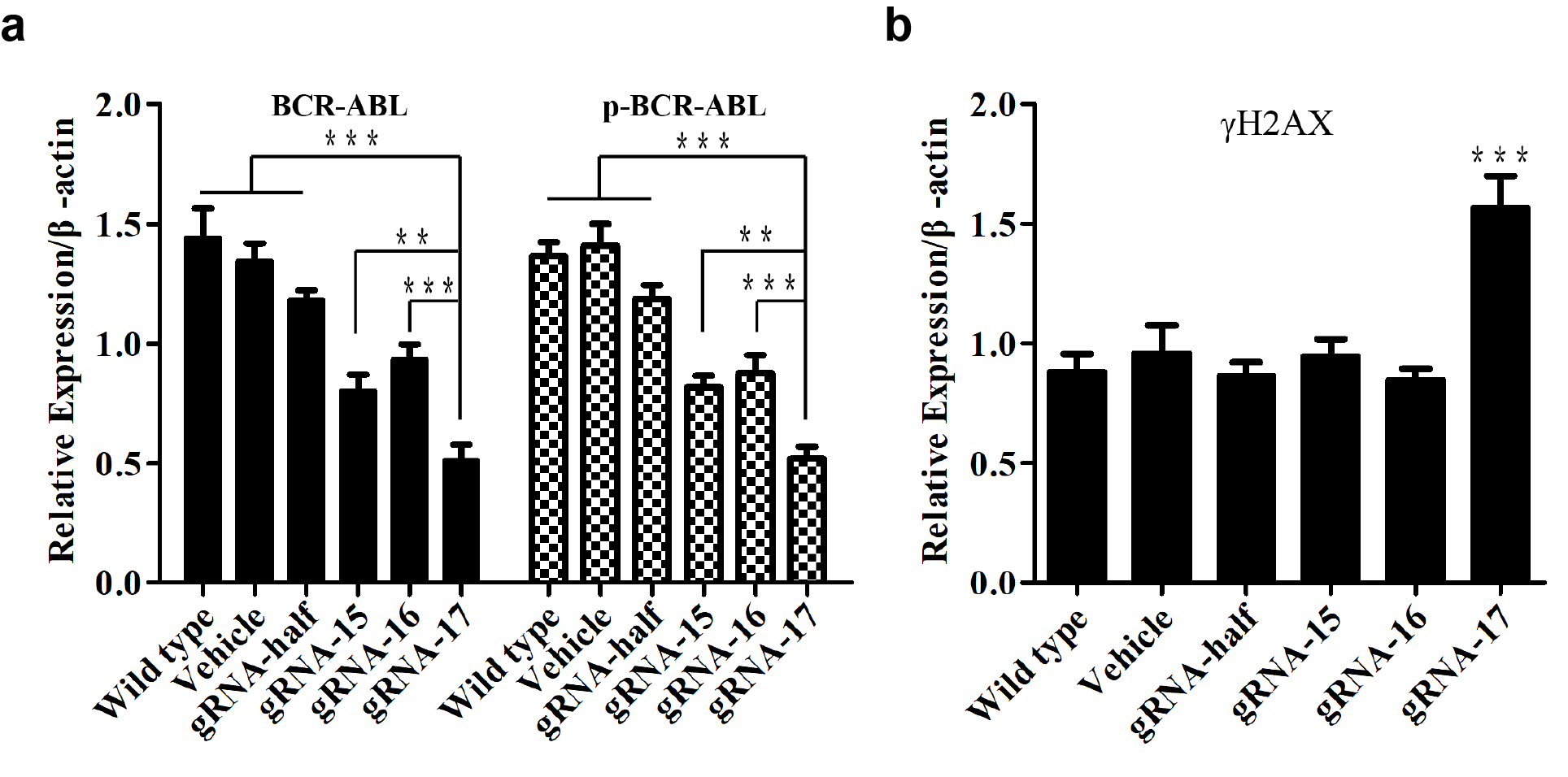

Supplement: Supplementary file 2 — Figure S1. Expressions of relative proteins were detected by western blot. K562 cells were treated with vehicle plasmids, RFNs-half plus donor, RFNs-15 plus donor (gRNA-15), RFNs-16 plus donor (gRNA-16), and RFNs-17 plus donor (gRNA-17), respectively. Non-transfected K562 cells were considered as wild type group. (a) The relative expressions of p-BCR-ABL and BCR-ABL normalized to β-actin were quantified. (b) The relative expressions of γH2AX normalized to β-actin were quantified. The results are presented as the means ± SD. p < 0 .01 (**) and p < 0.001 (***). (TIF 264 kb) [file 13046_2019_1229_MOESM2_ESM.tif]

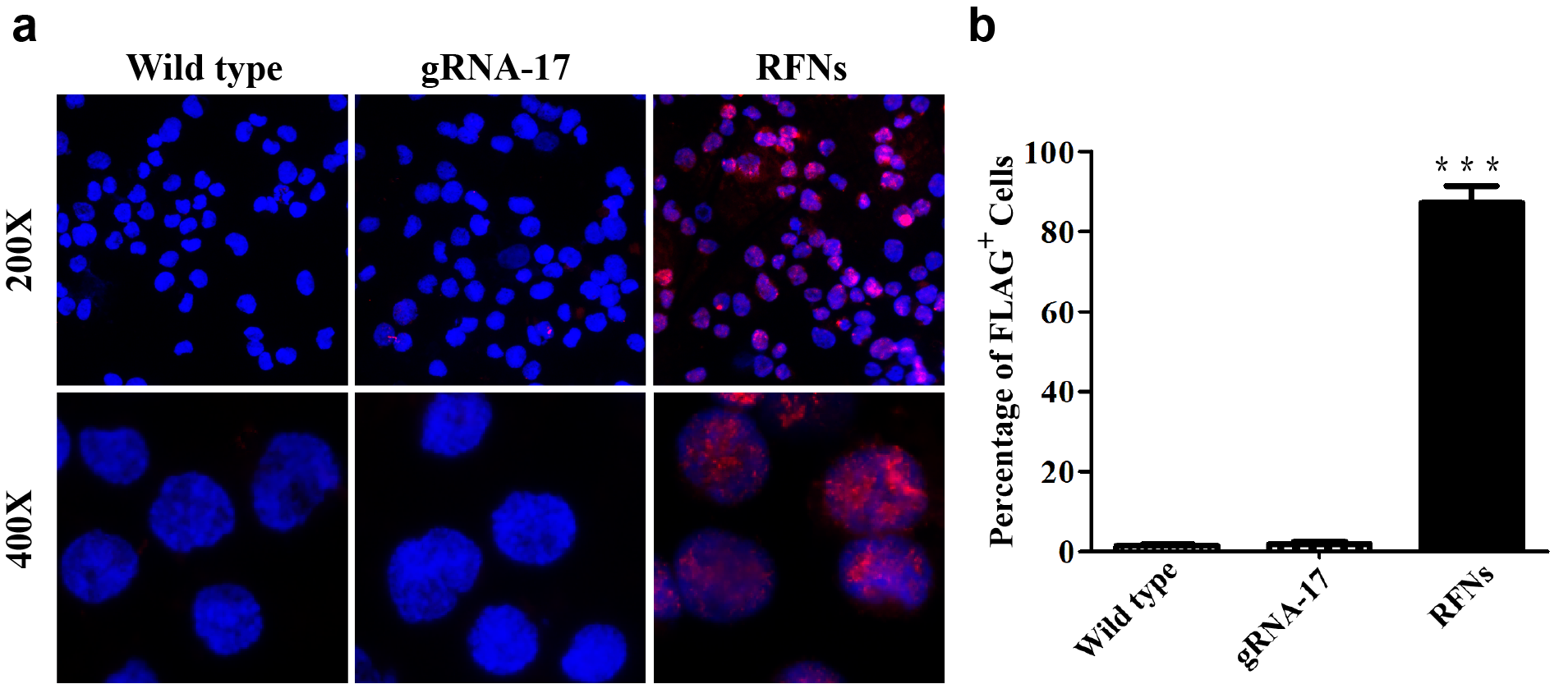

Supplement: Supplementary file 3 — Figure S2. The transfection efficiency of K562 cells was estimated by detection of FLAG tagged of FokI-dCas9. K562 cells were transfected with gRNA-17 and RFNs respectively. The untreated K562 cells were considered as negative control. (a) FLAG tag was detected by immunofluorescent assay after 48 h of transfection. (b) The percentage of FLAG positive cells was quantified by counting 300 cells in total. (TIF 1277 kb) [file 13046_2019_1229_MOESM3_ESM.tif]

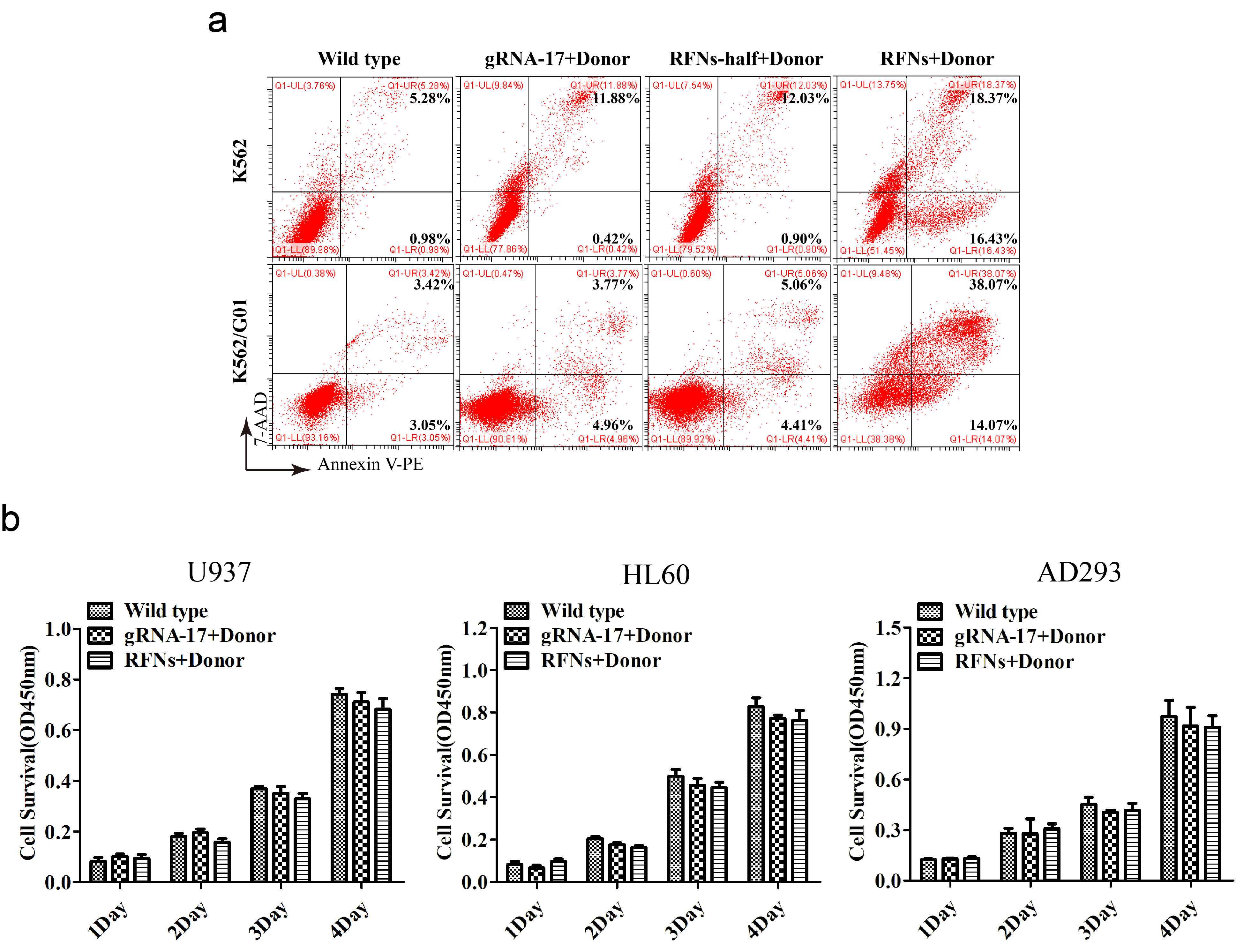

Supplement: Supplementary file 4 — Figure S3. (a) RFNs suppress viability and induce apoptosis of imatinib sensitive and resistant cells. Cells were transfected with gRNA-17 plus donor, RFNs-half plus donor, RFNs plus donor, respectively. The apoptotic rate of cells was analyzed by flow cytometry. (b) Cell viability of bcr-abl negative cells was tested via CCK-8 assay. U937, HL60, and AD293 cells were transfected with gRNA-17 plus donor or RFNs plus donor. (TIF 1306 kb) [file 13046_2019_1229_MOESM4_ESM.tif]

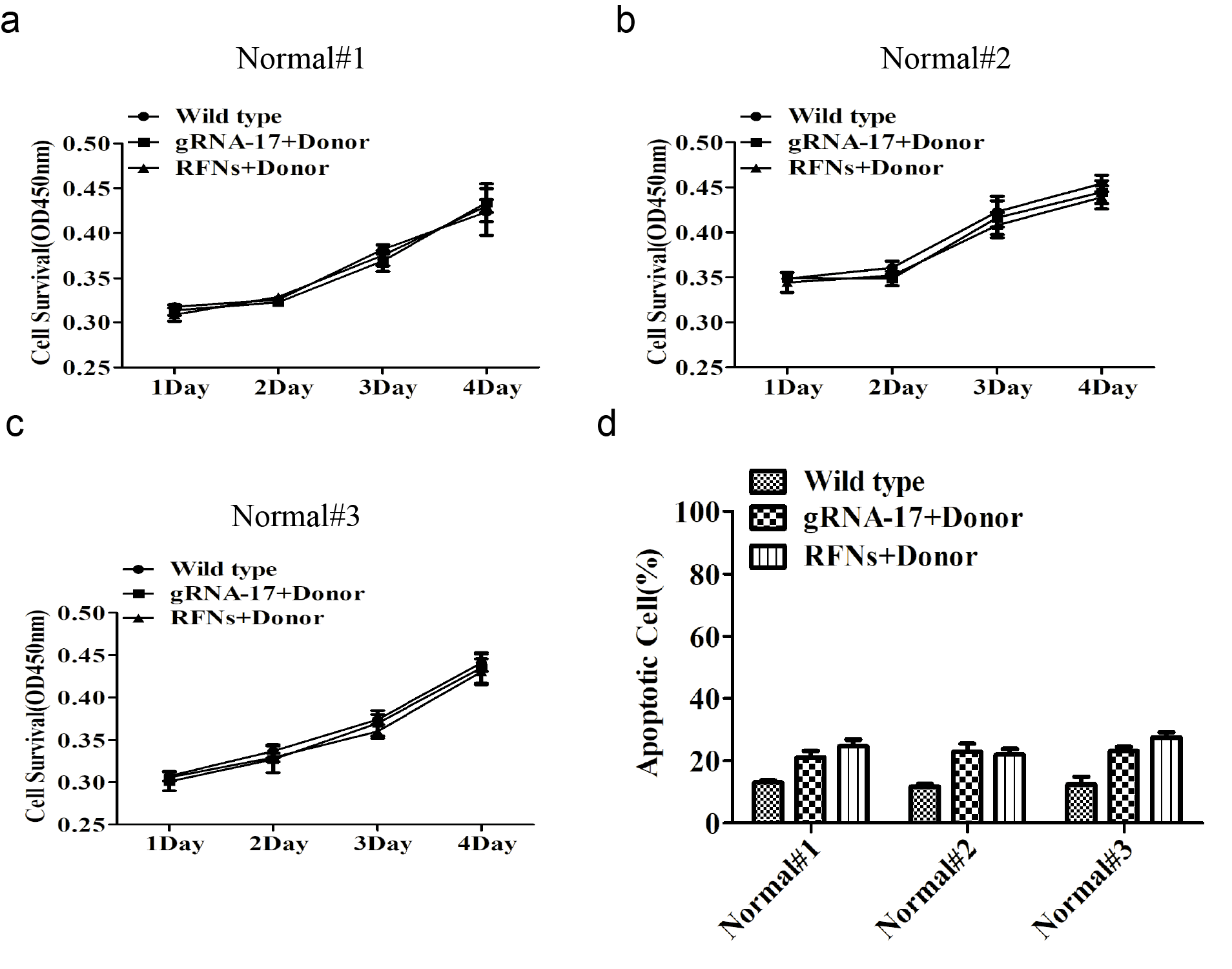

Supplement: Supplementary file 5 — Figure S4. RFNs have hardly any effect on the proliferation and apoptosis of bcr-abl negative CD34+ cells. The bcr-abl negative CD34+ cells were isolated from individuals diagnosed with leukocytosis or anemia, and transfected with gRNA-17 plus donor or RFNs plus donor. (a)-(c) Cell viability of normal CD34+ cells was detected by CCK-8 assay. (d) Apoptotic proportion of normal CD34+ cells was determined by flow cytometry. (TIF 413 kb) [file 13046_2019_1229_MOESM5_ESM.tif]

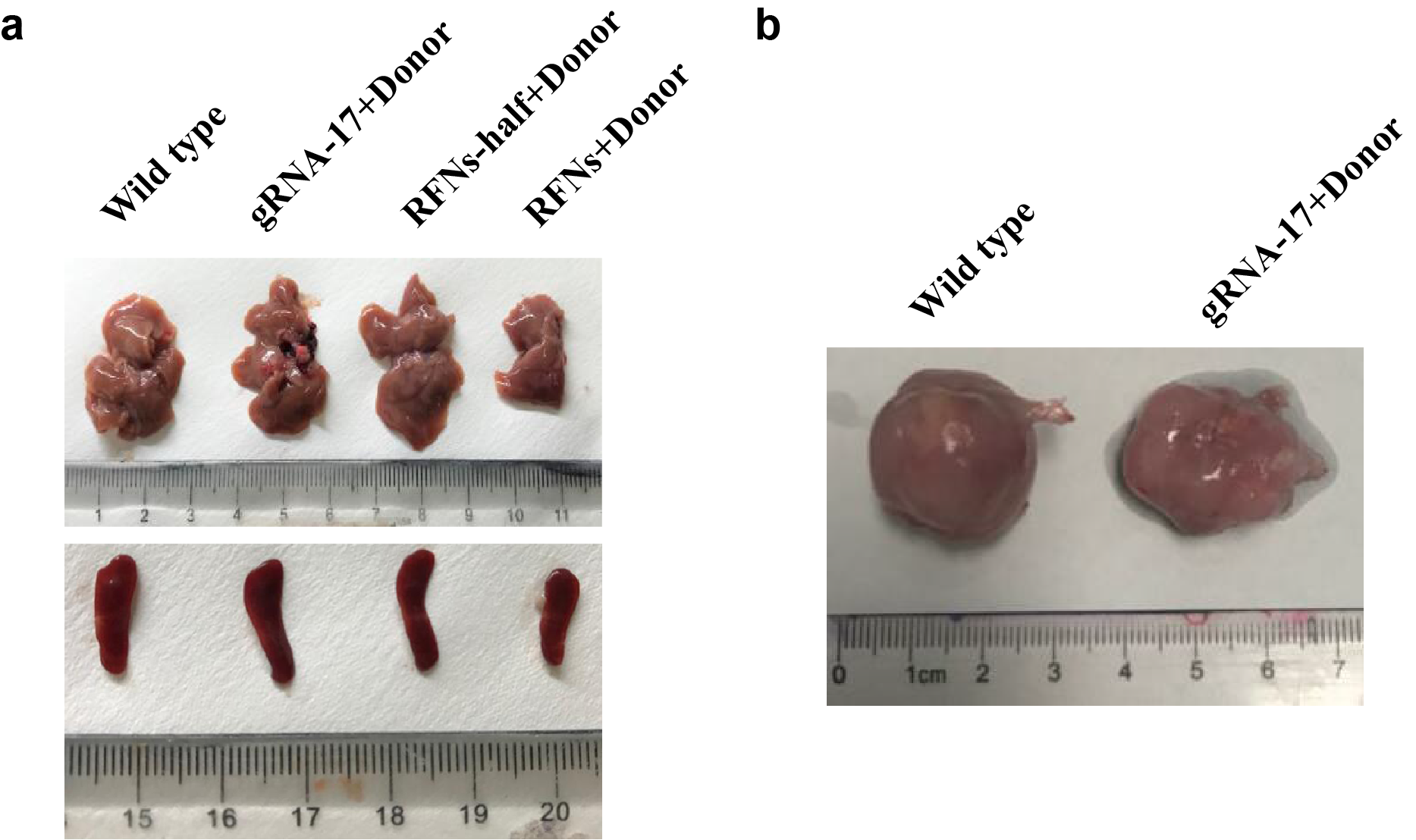

Supplement: Supplementary file 6 — Figure S5. (a) Typical pictures of spleen and liver from wild type group, gRNA-17 plus donor group, RFNs-half plus donor group and RFNs plus donor group. (b) Picture of part solid tumors from wild type group and gRNA-17 plus donor group. (TIF 2127 kb) [file 13046_2019_1229_MOESM6_ESM.tif]
